# Calcium, TRPC channels, and regulation of the actin cytoskeleton in podocytes: towards a future of targeted therapies

**DOI:** 10.1007/s00467-015-3224-1

**Published:** 2015-10-21

**Authors:** Nicolas Wieder, Anna Greka

**Affiliations:** Renal Division, Department of Medicine and Glom-NExT Center for Glomerular Kidney Disease and Novel Experimental Therapeutics, Brigham and Women’s Hospital and Harvard Medical School, Boston, MA USA; Broad Institute of MIT and Harvard, Cambridge, MA USA

**Keywords:** Calcium, TRPC channels, Podocytopathies, Steroid-resistant nephrotic syndrome, Glomerular disease, Angiotensin type 1 receptor (ATR1), Cytoskeleton, Children

## Abstract

With more than 6,000 new pediatric patients with treatment-resistant nephrotic syndrome in the US each year alone, the unmet need for novel, podocyte-specific therapies is substantial. Recently, the established therapeutic benefit of angiotensin-converting enzyme (ACE) inhibitors and angiotensin receptor blockers (ARB) was used as a starting point to gain insight into the pathomechanism of primary podocytopathies. A calcium (Ca^2+^)-mediated pathway has been identified that connects the angiotensin type 1 receptor (AT1R) to podocyte cytoskeletal dynamics, essential for a functioning glomerular filtration barrier. This discovery provided an important missing piece in our understanding of the pathomechanism of filter barrier damage, revealing Ca^2+^ signaling as critical for podocyte health and disease. The identification of the two Ca^2+^ permeant channels TRPC5 and TRPC6 as mediators of this pathway not only bolstered the importance of podocyte cytoskeleton dynamics but also revealed promising drug targets for treatment-resistant nephrotic syndrome. This review will focus on this novel signaling pathway in primary podocytopathies and its implications for next-generation therapies for glomerular disease.

## Case report

A 16-year-old boy who presented with nephrotic syndrome since the age of 7, with an initial biopsy showing minimal change disease (MCD), and with a subsequent biopsy showing primary FSGS (focal segmental glomerulosclerosis) at age 9, presented with relapsing steroid-resistant nephrotic syndrome. The patient had preserved renal function with an average serum creatinine 0.5 mg/dl. His medications included lisinopril 10 mg daily, simvastatin 40 mg daily, and furosemide as needed, titrated to symptoms. Cyclosporine A, cytotoxic therapy with cyclophosphamide, mycophenolate mofetil, and rituximab had been added to steroids at different times during his adolescent years, without success in achieving remission. At age 16, while in partial remission on high-dose oral steroids, and during a subsequent attempt to slowly taper the steroids, he experienced recurrence of proteinuria with a urinary protein/creatinine ratio (UPC) of 12. Multiple attempts to achieve remission with high-dose steroids were unsuccessful. A few weeks later, he was admitted to the hospital with anasarca that required intravenous diuretics. A repeat kidney biopsy confirmed the diagnosis of primary podocytopathy or FSGS. What further therapeutic interventions may be available to this patient with steroid-resistant nephrotic syndrome?

## Introduction

### A podocentric view of glomerular disease

One of the main tasks of the kidney is to deplete low molecular weight molecules from the plasma, while larger, vital proteins such as albumin are retained in the bloodstream [1]. The glomerulus thereby constitutes the body’s filtration barrier and is, in this context, viewed as the central functional entity of the kidney [2]. A well-coordinated interplay of mesangial cells, parietal cells, endothelial cells, and podocytes is required to maintain the microscopically and macroscopically highly organized structure of the glomerulus [3]. Disruption of this system leads to glomerular disease, a large group of kidney diseases, characterized by proteinuria [4].

Based on the underlying disease mechanism, we would like to reinforce a podocentric classification that distinguishes primary and secondary podocytopathies. While secondary podocytopathies are the consequence of well-defined inflammatory responses in a variety of immune-mediated glomerular diseases such as ANCA vasculitis, lupus nephritis, or other immune complex glomerulonephritides, where the podocyte may be “collateral damage” due to primary endothelial and immune complex-mediated injury, primary podocytopathies are caused, by definition, by podocyte-specific injury. Histologically, primary podocytopathies are a heterogeneous group of kidney diseases with a great variety of anatomical features. These range from profound structural changes in the podocyte cytoskeleton only detectable at the electron microscopic level (as in foot process effacement in the euphemistically named “minimal change” disease (MCD)) all the way to profound light microscopically detectable focal sclerosis (as in FSGS) and/or completely collapsed glomeruli (as in collapsing glomerulonephritis) [3]. This review will mostly be concerned with primary podocytopathies.

The filtration barrier is composed of the fenestrated endothelium of the glomerular vessels, the glomerular basement membrane (GBM), and the interdigitating podocyte foot processes (FPs). Podocyte structures called slit diaphragms (SD) function like modified adherens junctions connecting neighboring FPs [5]. Through human genetic studies [3], a set of well-characterized proteins (such as nephrin, podocin, and others) have been identified in the SD as critical for podocyte–podocyte adhesion as well as signaling from the SD to the actin cytoskeleton [3]. Podocyte cell bodies float in the urinary space inside Bowman’s capsule, fixed only through their FPs to neighboring podocytes and through focal adhesions to the GBM [6]. Upon podocyte stress or injury, podocytes undergo a set of canonical structural changes, including foot process effacement (FPE) [6]. It has been proposed that FPE is a compensatory, adaptive mechanism for podocytes to avoid detachment [6]. Failure to maintain attachment appears to trigger mechanisms that lead to podocyte death, the hallmark of advanced glomerular disease [3]. Based on the essential role of the podocyte slit diaphragms as part of the renal filtration barrier [1] and podocyte cytoskeleton rearrangements as early compensatory responses to podocyte injury [6], podocytes have been rightly placed at the center of ongoing investigations into the pathomechanism of glomerular disease [3, 4].

Understanding the importance of the podocyte cytoskeleton for a functioning renal filter barrier poses a fundamental question: What is the underlying mechanism of cytoskeletal rearrangements in podocytes? Starting from the established benefit of angiotensin-converting enzyme (ACE) inhibitors and angiotensin receptor blockers (ARB) as our first-line, standard-of-care therapy for glomerular disease [7], and asking what mediates their anti-proteinuric, podocyte-specific effect, has revealed an interesting answer: a Ca^2+^-mediated pathway connects the angiotensin type 1 receptor (AT1R) to podocyte cytoskeletal dynamics. This pathway indeed provided an explanation for an important missing piece in our understanding of the pathomechanism of filter barrier damage, revealing Ca^2+^, a central player of the intracellular signaling apparatus in any cell type, as critical for podocyte health and disease. This review will focus on this novel signaling pathway and its implications for next generation therapies for glomerular disease.

## Calcium signaling

Ca^2+^ is a vital second messenger in virtually every cell type [8]. It orchestrates a great variety of different cellular functions such as muscle contraction [9], fertilization [10], neurotransmitter release [11], cytoskeleton dynamics [12] and apoptosis [13, 14]. Ca^2+^ signaling relies on a low intracellular Ca^2+^ resting concentration [Ca^2+^]_i_ (0.1 μM), roughly four orders of magnitude below the extracellular Ca^2+^ concentration [Ca^2+^]_e_ (2 mM). A Ca^2+^ signal is induced by an increase of the [Ca^2+^]_i_, whereby the major sources of Ca^2+^ are the extracellular space and intracellular Ca^2+^ stores such as the endoplasmic reticulum (ER) and the mitochondria [15]. Specialized ion channels, pumps, and cytosolic Ca^2+^ buffers constitute the cellular Ca^2+^ regulatory apparatus. Their well-coordinated interplay allows both the induction of intracellular Ca^2+^ signals (ON mechanisms) and the relaxation of the system towards its vital low resting [Ca^2+^]_i_ (OFF mechanisms) [16]. ON mechanisms are usually mediated by ion channels which, upon opening, allow Ca^2+^ to diffuse along its concentration gradient into the cytosol. Common examples include voltage-dependent Ca^2+^ channels (VDCCs) and intracellular ligand-gated Ca^2+^ release channels, such as the inositol 1,4,5-trisphosphate receptor (IP_3_R) or the ryanodine receptor (RYR), predominantly located on the ER membrane [15, 16]. OFF mechanisms, by contrast, extrude Ca^2+^ from the cytosol, thus pumping Ca^2+^ against its concentration gradient either into the extracellular space or into intracellular Ca^2+^ stores. Important examples are the sarco/endoplasmic reticulum Ca^2+^-ATPase (SERCA) and the plasma membrane Na^+^/Ca^2+^-exchanger (NCX) [15, 16]. Upon elevated [Ca^2+^]_i_, Ca^2+^ binds to highly conserved Ca^2+^ binding domains, such as EF-hand domains [17]. By introducing its two-fold positive charge, Ca^2+^ induces a conformational change in these proteins, which can lead to an alteration of their function. Ca^2+^-sensing proteins are functionally highly diverse and range from transcription factors and phosphatases [8] to elements of the cytoskeleton [12] and proteins of the contractile apparatus in muscle cells [18]. A prolonged increase in [Ca^2+^]_i_ induces cell death [13, 14] and thus Ca^2+^ signals are required to be temporally limited. Furthermore, to be able to mediate the great variety of Ca^2+^-dependent cellular functions, Ca^2+^ signals are not necessarily binary signals, but can also encode information in a frequency-dependent manner (for example, as Ca^2+^ oscillations) [18]. Spatial heterogeneity is another important aspect to understand the high specificity of Ca^2+^ signals. Instead of looking at the cell as a single homogeneous system, it is necessary to identify so-called Ca^2+^ microdomains, sub-femto liter domains that accumulate important Ca^2+^ signaling elements. Such microdomains can be predetermined by structural features of the cell, such as dendritic spines in neurons [19] or FPs in podocytes [20], or their spatial extent can be limited by diffusion and the cytosolic Ca^2+^ buffering capacity. Acknowledgment of the importance of Ca^2+^ microdomains to our understanding of Ca^2+^ signaling in general is constantly increasing [21], which is reflected in a growing number of theoretical studies investigating the mathematical foundations of the complex non-linear dynamics of Ca^2+^ microdomains [22–25].

## TRPC channels and calcium signaling in podocytes

**Fig. 1:**
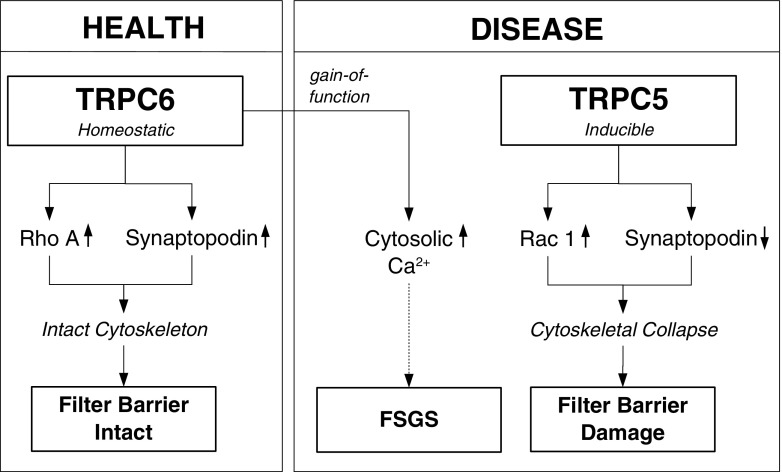
Simplified schematic presentation of our current understanding of TRPC channels in podocytes in health and disease. TRPC6 has a homeostatic function, maintaining a functioning podocyte cytoskeleton through RhoA activation and thus an intact filter barrier. Increased activity (e.g., gain-of-function mutations) disrupts cytosolic Ca^2+^ homeostasis and is known to cause focal segmental glomerulosclerosis (FSGS). In contrast, TRPC5 is inducible and activates Rac1, which has been shown to cause cytoskeletal collapse and consecutive filter barrier damage

Transient receptor potential (TRP) channels are a heterogeneous group of non-selective cationic channels, containing 28 human homologs, subdivided into seven subfamilies [26, 27]. Two members of the canonical TRP (TRPC) channel subfamily have been identified to play a major role in the Ca^2+^ homeostasis of podocytes, namely TRPC6 and TRPC5 [28] (Fig. 1). Human genetics has revealed TRPC6 as a vital component of the slit diaphragm [29], and further investigation into gain-of-function TRPC6 mutations ultimately led to the identification of TRPC5 as a critical inducible mediator of Ca^2+^-induced cytoskeletal remodeling in podocytes [28], an important event at the onset of all podocytopathies [30, 31].

## Angiotensin II-mediated Ca^2+^ signals

The beneficial therapeutic effects of renin–angiotensin–aldosterone system (RAAS) inhibition in primary podocytopathies are well established [32]. However, molecular insights into the underlying mechanisms were lacking until the AT1R was identified as the upstream receptor signaling to both TRPC5 and TRPC6 in podocytes [28]. The Gq subunit of the G-protein-coupled AT1R activates phospholipase C (PLC), which in turn controls TRPC5 /TRPC6 gating. While the detailed mechanisms for differential and specific activation of each channel have not yet been fully elucidated, the activation of both channels leads to Ca^2+^ influx into podocytes, with distinct cellular responses [28].

Tian et al. showed that while TRPC6 activates RhoA, inducing a stationary, homeostatic podocyte phenotype, TRPC5 activates Rac1 inducing a disease-associated motile phenotype: in other words, triggering a cellular program that centers on Rac1 to mediate maladaptive cytoskeletal remodeling leading to FPE [28, 30]. Indeed, prior to the work revealing TRPC5/Rac1 and TRPC6/RhoA coupling, the small Rho GTPases (RhoA, Rac1, and Cdc42) had been associated with different aspects of cell motility and cytoskeleton dynamics in podocytes [31], but they had not been linked to the TRPC channels or to Ca^2+^ signaling.

However, how can individual TRPC channels mediate distinct cellular events? A unifying explanation can be found in the existence of Ca^2+^ microdomains in podocytes, to which each TRPC channel introduces small amounts of Ca^2+^, locally increasing [Ca^2+^]_i_, sufficient to activate a localized effector [28]. Thus, both TRPC5 and TRPC6 are essential for a functioning podocyte cytoskeleton: gene silencing of TRPC6 induces the activation of Rac1, the loss of stress fibers and increased podocyte motility, in summary a motile phenotype. Constitutive activation of RhoA rescues the phenotype [28]. Interestingly, similar to gain-of-function mutations in humans, overexpression of TRPC6 in podocytes in vitro also leads to loss of stress fibers in podocytes [33] and proteinuria in mice [34], indicating the necessity of a well-balanced TRPC6 activity to maintain a healthy podocyte cytoskeleton (Fig. 1). Conversely, gene silencing of TRPC5 induces stress fiber formation and RhoA activation and decreases podocyte motility, and thus leads to a stationary phenotype. In this case, constitutive activation of Rac1 rescues the phenotype [28]. In summary, these findings suggested that instead of the activity of one particular pathway in podocytes, it is the balanced activity of both TRPC6/RhoA and TRPC5/Rac1 that is critically important. Subsequent work in vivo corroborated these findings and further showed that, in the setting of early proteinuric disease, TRPC5 is induced to activate Rac1 leading to FPE. Most importantly, this could be blocked by a small molecule inhibitor, ML204, in a dose-dependent fashion. This work revealed a new, ion channel-targeted approach to anti-proteinuric therapy [30].

## Calcineurin signaling

The Ca^2+^-dependent phosphatase calcineurin is typically known for activating the transcription factor nuclear factor of activated T-cell (NFAT) in T-cells, upregulating interleukin 2 (IL-2), and thus inducing the T-cell response [35]. However, calcineurin has also been shown to have a distinct role in podocytes. Calcineurin activation leads to cathepsin L-mediated cleavage of the vital, actin-associated protein synaptopodin [36], which plays an essential role in podocyte cytoskeletal homeostasis [37]. Activation of calcineurin induces synaptopodin cleavage and proteinuria, while the calcineurin inhibitor cyclosporine A (CsA) prevents it [38]. This finding challenged the idea that the treatment of proteinuric kidney diseases with CsA is based on immunomodulation, and emphasized instead the importance of an intact podocyte cytoskeleton for the glomerular filtration barrier [3], revealing the podocyte as the therapeutic target of choice for glomerular diseases.

The fact that FSGS-causing TRPC6 gain-of-function mutations activate calcineurin-NFAT-dependent gene transcription [39] further points towards an important role of TRPC channels and calcineurin signaling in podocytes. In cardiac hypertrophy, the calcineurin-NFAT pathway induces the upregulation of TRPC6 transcription [40] and, as just recently proposed, a similar mechanism appears to exist in podocytes [41]. Perhaps this mechanism constitutes an auto-regulatory positive feedback loop, eventually increasing the presence of TRPC6 channel density on the plasma membrane. Further investigations need to be directed towards the understanding of this mechanism to elucidate the full scope of TRPC channel-induced podocytopathies.

## Discussion

How do the experimental findings summarized here translate into clinical medicine? One way to evaluate the significance of these insights is to look at the consequences of mutations in key molecules of the proposed signaling pathway. Mutations of TRP channels have been associated with a number of different diseases. Such TRP channelopathies include night blindness (TRPM1) [42], progressive familial heart block type I (TRPM4) [43], hypomagnesemia with secondary hypocalcemia (TRPM6) [44], and autosomal dominant polycystic kidney disease (TRPP2) [45], to name a few. In 2005, mutations in TRPC6 were identified in several forms of hereditary FSGS [29, 46].

At that time, proteinuric kidney disease was primarily associated with rearrangements in the cytoskeletal structure of podocytes, thus the identification of a Ca^2+^ channel causing FSGS initially suggested the potential for a new pathomechanism [46]. However, by connecting TRPC6 activation to podocyte cytoskeletal remodeling, we in fact gained further evidence to bolster the view of impaired cytoskeletal dynamics as the central pathomechanism of early proteinuric kidney disease [28]. Just recently, the important role of TRPC6 in this context was highlighted in a mouse model with constitutively active Gqα subunits in podocytes [41]. The consequent increased activation and upregulation of an ion channel identified as TRPC6 was shown to induce albuminuria and FSGS, as well as a decrease in the number of glomerular podocytes [41]. This study did not investigate a role for TRPC5 in this mouse model, but despite the lack of these specific data, the fact that orthogonal approaches continue to implicate TRPC channels in the emergence of FSGS further bolsters the idea that TRPC channels and Ca^2+^-mediated pathways may be excellent therapeutic targets for a disease that currently lacks effective treatment options.

Drugs targeting ion channels are in widespread clinical use: common representatives are verapamil (voltage-gated L-type Ca^2+^ channel blocker), lidocaine (voltage-gated Na^+^ channel blocker) and diazepam (GABA A, ligand-gated Cl^−^ channe6l) [47]. Historically, early attempts at drug targeting of ion channels had limited success due to lack of specificity and significant off-target effects, which limited their applicability [48]. However, in the last decades, great progress has been made in our understanding of ion channel structures and function, and consequently we have developed drugs with greater specificity and fewer off-target effects [47]. Modern techniques for systematic high-throughput drug screening have also made the identification of new compounds more feasible.

Given the orthogonal convergence of human genetics (TRPC6), the cell biology of the actin cytoskeleton (RhoGTPases) and our new knowledge of Ca^2+^ physiology in podocytes (TRPC5 and TRPC6), we may now be poised to uncover new treatment options for primary podocytopathies based on the modulation of TRPC5 and TRPC6 channel activity.

A first promising step in this direction was the identification of ML204, a small molecule inhibitor of TRPC4/5 channels [49]. Since TRPC4 is not expressed in podocytes [30], whereas TRPC5 has been detected at the single channel level by electrophysiology studies in podocytes [28], the effect of ML204 on podocyte TRPC5 became the focus of investigation. Combining experiments in TRPC5 knockout mice, ex vivo physiology in isolated glomeruli, in vitro real-time live imaging of the podocyte cytoskeleton, the lipopolysaccharide (LPS) and the protamine sulfate (PS) models of primary podocytopathies in vivo, TRPC5 was found to be the upstream regulator of LPS-induced albuminuria and PS-induced filter barrier disruption [30]. Based on these data, disease-induced upregulation of TRPC5 with a consequent disruption of the balance between the RhoA and Rac1 pathways in favor of Rac1, suggests that a TRPC5-specific inhibitor must be able to restore physiologic (TRPC6 and RhoA-mediated) Ca^2+^ homeostasis in podocytes. Indeed, the small molecule inhibitor ML204 was not only shown to be an effective pore blocker of TRPC5-mediated Ca^2+^ transients in podocytes, but it was also shown to rescue podocytes from disease-associated cytoskeletal remodeling. This effect was further validated with Ca^2+^ imaging in isolated glomeruli, emphasizing the physiological relevance of the in vitro model in podocytes [30]. These data, which await validation in other models of progressive proteinuric kidney disease, emphasize the central role of TRPC5 in primary podocytopathies and raise hope that targeting TRPC5 may indeed ultimately offer a novel treatment option for glomerular diseases caused by the disruption of the podocyte cytoskeleton.

The finding that gain-of-function mutations in TRPC6 cause familial FSGS [46] has also provided an excellent rationale for significant effort in identifying potent TRPC6 inhibitors as a therapeutic approach to podocytopathies. A number of inhibitors have indeed been developed, some with a favorable specificity profile for TRPC6 over its close homologs TRPC3 and TRPC7 [50]. However, careful consideration of an important nuance about TRPC6 may, and probably should, somewhat diminish enthusiasm for drugs targeting TRPC6: careful cell biological and physiological analysis by a number of groups has shown that TRPC6 plays an important homeostatic role in podocytes, as it is essentially important for maintenance of RhoA signaling and cytoskeletal integrity [28, 51]. In our understanding, blocking or abrogating an essential homeostatic mechanism in podocytes by blocking homeostatic TRPC6 activity is likely to lead to the opposite of the intended therapeutic effect: we expect it to actually cause or worsen FPE and proteinuria. By this scenario, it is only in the event of gain-of-function mutations in TRPC6 (or perhaps significantly enhanced channel activity or upregulation) where a TRPC6 blocker may be effective to dampen or block the injurious excess activity (which translates into excess Ca^2+^-mediated toxicity and cell death [52–55]). Therefore, this may be a case in which a simple, initial glance at the genetics might seemingly point in the direction of blocking TRPC6, but a nuanced understanding of the physiology and cell biology may suggest that alternative strategies should be sought, ones that only attack inducible, and not homeostatic, Ca^2+^ influx.

Identification of the Ca^2+^-regulated phosphatase calcineurin as an important link between TRPC/Ca^2+^ signaling and cytoskeletal dynamics in podocytes [20, 28] introduced a new view on the mechanism of action of cyclosporin A (CsA) in FSGS and other glomerular diseases. Instead of acting through its classical immunologic effect on NFAT signaling in T-cells, it appears to inhibit calcineurin in a podocyte-specific manner to protect the podocyte homeostatic structural protein synaptopodin from dephosphorylation and ultimate degradation [38]. Considering the unfavorable spectrum of kidney-specific side effects of long-term use of CsA, including hyalinosis and interstitial fibrosis, direct targeting of podocyte-specific calcineurin substrates such as synaptopodin may offer a good alternative strategy for the development of novel anti-proteinuric drugs with podocyte-specific CsA-like effects [38]. Specifically, a synaptopodin protective agent may in fact be an excellent therapeutic strategy.

The emerging importance of the essential role of an intact podocyte cytoskeleton for a functioning filtration barrier has been further bolstered by the identification of B7-1 (CD80), as yet another potentially druggable component of the regulatory apparatus of the podocyte cytoskeleton. B7-1 was shown to be upregulated upon podocyte injury [56], thus preventing podocyte beta 1 integrin activation, and eventually leading to cytoskeletal remodeling in podocytes in vitro and in vivo [56]. The biologic abatacept has traditionally been used for the treatment of rheumatoid arthritis (RA) [57], where it acts as a T-cell inhibitor by binding to CD80 (B7-1) and CD86, thus blocking the costimulatory receptor CD28 [58]. Understanding of the role of B7-1 as a T-cell-independent, podocyte-specific cytoskeleton regulator motivated a proof of concept study, in which abatacept was used as a treatment in five patients with treatment-resistant nephrotic range proteinuria [56]. Abatacept induced remission in all five patients and thereby impressively confirmed the central role of the podocyte cytoskeleton in glomerular disease in general and the pathophysiological significance of B7-1 in particular.

## Conclusions

The hallmark of primary podocytopathies is proteinuria, a direct consequence of filter barrier damage. Functionally, podocytes are critical for maintaining an intact filtration barrier [3]. Importantly, podocytes are terminally differentiated cells, quite similar to neurons, and therefore their capacity for regeneration is limited. Podocytes have developed potent “adaptive” mechanisms to cope with acute and chronic stress and injury. Also similar to neurons, podocytes have exquisite mechanisms for the tight control of Ca^2+^ homeostasis, because the consequence of excess Ca^2+^ is cytoskeletal collapse [30, 52–55] without the possibility of a suitable replacement. In this scenario, FPE may be seen as an initial (futile) attempt to maintain a (filtration) barrier in the face of injury. Clinically, this manifests as proteinuria, and precedes the presence of histological sclerotic lesions, which are the result of podocyte death and maladaptive proliferation of parietal epithelial cells on the denuded GBM [59].

**Fig. 2:**
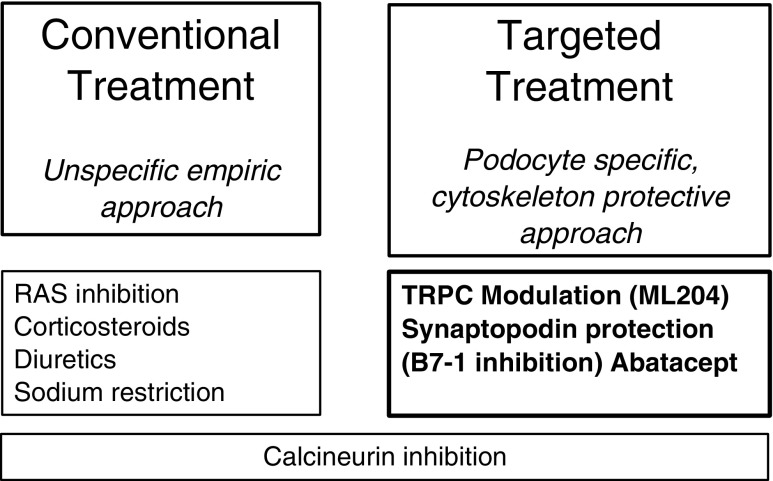
Current standard treatment options for primary podocytopathies (Conventional Treatment) and podocyte-targeted, cytoskeleton protective treatment (Targeted Treatment). Calcineurin inhibitors have been used as second-line therapy for corticosteroid-resistant FSGS on an empiric basis. The finding that calcineurin inhibitors prevent synaptopodin cleavage makes it also a cytoskeleton protective agent

The identification of TRPC5 and TRPC6 as upstream regulators of Ca^2+^-mediated cytoskeletal rearrangements in podocytes, and the notion of maintaining a proper balance between homeostatic TRPC6/RhoA versus inducible TRPC5/Rac1 activity constitutes a novel paradigm for understanding filter barrier damage in proteinuric diseases. Together with the development of improved TRPC channel inhibitors, these findings constitute a new approach to the unmet need of effective treatment options for primary podocytopathies such as MCD, FSGS, and diabetic kidney disease, as well as other acquired proteinuric kidney diseases. Furthermore, new drugs interacting with downstream targets of calcineurin, most importantly synaptopodin, could constitute a treatment option that is, due to an optimized side effect spectrum, more attractive than the currently used CsA (Fig. 2).

Patients with initial MCD diagnoses, resistant to steroid treatment, with subsequent biopsies showing FSGS, as described in the case report at the beginning of this review, appear frequently in the renal clinic. Following the here-proposed mechanistic understanding of the pathogenesis of primary podocytopathies, the seemingly distinct entities we call MCD and FSGS may not necessarily be two different diseases, but rather the transition from an initial state of a collapsed cytoskeleton (MCD) to a decompensated state of permanent podocyte loss and replacement with scar (FSGS). This new, molecularly-defined understanding of the mechanisms underlying primary podocytopathies also brings into crisper focus the therapeutic potential of targeting the podocyte cytoskeleton.

With more than 6,000 new pediatric patients suffering from treatment-resistant nephrotic syndrome in the US each year alone [60], the unmet needs of patients with treatment-resistant nephrotic syndrome is substantial, and calls for novel, podocyte-specific therapies. While first results are promising, further studies are necessary to advance towards a future when clinicians will have at their disposal an array of molecular targeted, precision medicine strategies for primary podocytopathies.
